# Cerebral malaria – modelling interactions at the blood–brain barrier *in vitro*

**DOI:** 10.1242/dmm.049410

**Published:** 2022-07-11

**Authors:** Yvonne Adams, Anja Ramstedt Jensen

**Affiliations:** Centre for Medical Parasitology at the Department of Immunology and Microbiology, Faculty of Health and Medical Sciences, University of Copenhagen, 2200 Copenhagen N, Denmark

**Keywords:** Blood–brain barrier, *In vitro* models, Malaria, *Plasmodium falciparum*

## Abstract

The blood–brain barrier (BBB) is a continuous endothelial barrier that is supported by pericytes and astrocytes and regulates the passage of solutes between the bloodstream and the brain. This structure is called the neurovascular unit and serves to protect the brain from blood-borne disease-causing agents and other risk factors. In the past decade, great strides have been made to investigate the neurovascular unit for delivery of chemotherapeutics and for understanding how pathogens can circumvent the barrier, leading to severe and, at times, fatal complications. One such complication is cerebral malaria, in which *Plasmodium falciparum-*infected red blood cells disrupt the barrier function of the BBB, causing severe brain swelling. Multiple *in vitro* models of the BBB are available to investigate the mechanisms underlying the pathogenesis of cerebral malaria and other diseases. These range from single-cell monolayer cultures to multicellular BBB organoids and highly complex cerebral organoids. Here, we review the technologies available in malaria research to investigate the interaction between *P. falciparum*-infected red blood cells and the BBB, and discuss the advantages and disadvantages of each model.

## Abstract (precis)

**Summary:** This Review discusses the available *in vitro* models to investigate the impact of adhesion of *Plasmodium falciparum*-infected red blood cells on the blood–brain barrier, a process associated with cerebral malaria.

## Introduction

The protozoan parasite *Plasmodium falciparum* is the most prevalent malaria-causing species and remains a global health concern, with 241 million people reported infected and over 647,000 fatalities in 2020 (World Health Organization, 2021). Sub-Saharan Africa continues to bear the greatest burden, with 95% of both cases and fatalities occurring on the continent and children under the age of 5 years accounting for 74% of the global fatalities. The malaria parasite is capable of infecting red blood cells and, upon doing so, modifying their surface to express highly variable proteins called *P. falciparum* erythrocyte membrane protein 1 (*Pf*EMP1). The parasite uses these *Pf*EMP1 proteins to evade destruction via the spleen, by anchoring infected red blood cells (iRBCs) to the lining of the blood vessels via a variety of receptors expressed by human endothelial cells. This binding is termed sequestration. Receptors such as intercellular adhesion molecule 1 (ICAM-1), endothelial protein receptor C (EPCR, also known as PROCR) and CD36, and the sulphated glycosaminoglycan chondroitin sulphate A (CSA) have all been shown to have clinical significance in iRBC sequestration (Baruch et al., 1997; Cooke et al., 1994; Reeder et al., 1999; Turner et al., 2013). As an example, CSA is associated specifically with pregnancy-associated malaria, whereas the dual binding of ICAM-1 and EPCR to *Pf*EMP1 has been linked to cerebral malaria, the most severe form of the disease that is frequently fatal (Lennartz et al., 2017).

Cerebral malaria is characterised by the accumulation of iRBCs within the cerebral microvasculature, leading to vessel occlusion, loss of tight junction integrity and rapid-onset brain swelling, which can lead to death due to herniation of the brain (Crawley et al., 1998; Newton et al., 1991; Potchen et al., 2018; Seydel et al., 2015). Despite recent progress, the pathogenesis of cerebral malaria is not completely understood, mostly due to a lack of access to human tissues and paucity of representative animal models (Craig et al., 2012). Post-mortem studies from fatal cases of cerebral malaria have shown disruption of the blood–brain barrier (BBB). This key step in cerebral malaria occurs via alterations in the expression levels of tight junction proteins, such as occludin (OCLN) and ZO-1 (also known as TJP1) (Brown et al., 1999). Additionally, *ex vivo* analysis of serum or cerebrospinal fluid samples from patients confirms BBB dysfunction (Brown et al., 1999, 2001; Thakur et al., 2018).

Until recently, *P. falciparum-*iRBCs were not thought to cross the BBB; however, our group demonstrated that specific parasites that utilise ICAM-1 and EPCR simultaneously not only entered brain endothelial cells, but were also able to cross the BBB (Adams et al., 2021; Lennartz et al., 2017). Using a complex three-dimensional BBB organoid model (described in more detail below), we demonstrated that dual-receptor-binding parasites can induce swelling of the organoids, whereas non-dual-receptor-binding parasites do not (Adams et al., 2021). The presence of iRBCs within endothelial cells was verified in post-mortem samples from fatal cases of cerebral malaria. The literature has scant reports of perivascular iRBCs in humans (Edington, 1954; Janota and Doshi, 1979; Pongponratn et al., 2003), although this phenomenon is more prevalent in mice infected with the rodent malaria parasite *Plasmodium chabaudi*, which are commonly used murine models for malaria (Mota et al., 2000).

Understanding the impact of iRBC binding to the cerebral microvasculature is crucial, not only for the development of new chemotherapeutics and adjunctive treatments to minimise the long-term complications following cerebral malaria, but also to increase the molecular understanding of the pathological processes initiated by host–parasite interactions. To achieve this, robust models of the BBB are required. Here, we review and evaluate the available BBB models and technologies that allow researchers to investigate the interactions between *P. falciparum*-iRBCs and the BBB.

## Heterogeneity of endothelial beds

**Fig. 1.**
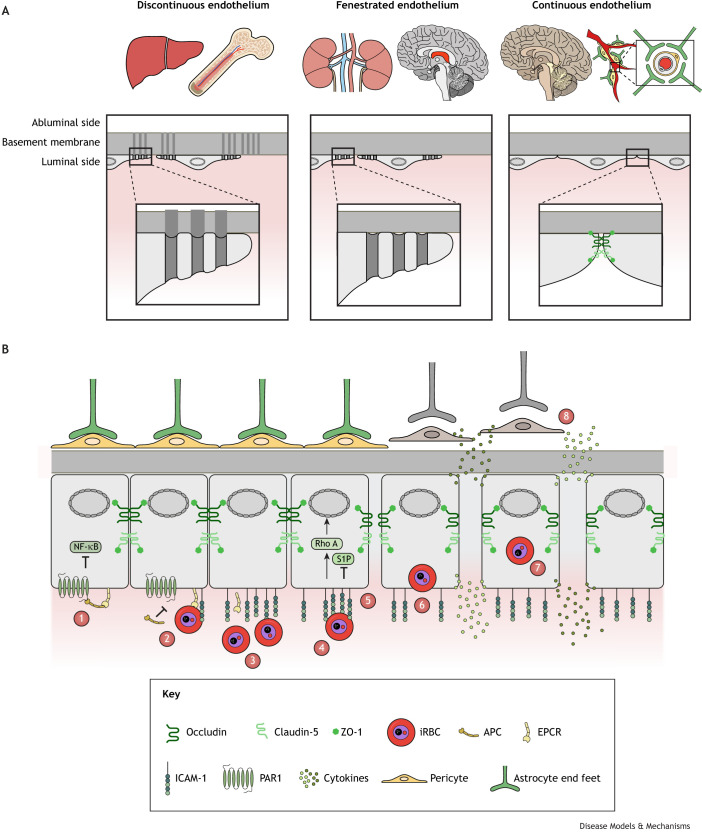
**Types and functions of the endothelium.** (A) Schematic of different types of endothelia found in the human body. Discontinuous endothelia are found in the liver sinusoids and bone marrow, and feature larger openings in the cells, typically 60-180 nm in diameter, which extend through the basement membrane. Fenestrated endothelia are found in the glomeruli of the kidneys and the choroid plexus in the brain (red). The fenestrations (pores) within the endothelial cells are typically 60-80 nm in diameter. Continuous endothelia form the blood–brain barrier (BBB) and are characterised by tight junctions between cells and highly selective transport of molecules. (B) The integrity of the continuous endothelium of the BBB can be compromised when *P. falciparum-*iRBCs bind to endothelial cell surface receptors. (1) In healthy endothelia, APC can bind to PAR1 (also known as F2R), inhibiting NF-κB and ensuring a cytoprotective state. ZO-1, claudin-5 and occludin combine to form tight junctions to help maintain barrier integrity. (2) iRBCs capable of binding to both ICAM-1 and EPCR outcompete APC, triggering a pro-inflammatory state within endothelial cells, leading to a loss of EPCR surface expression (Moxon et al., 2013). (3) iRBCs can bind to ICAM-1, which is upregulated in malaria (Tripathi et al., 2006) and (4) which clusters around bound iRBCs (Adams et al., 2021). (5) This binding to ICAM-1 and clustering inhibits the production of sphingosine-1-phosphate (S1P) whilst triggering Rho A, which induces NF-κβ. Combined, this results in a loss of the tight junctions and barrier integrity. This is exacerbated further when (6) iRBCs enter the endothelial cells, further destabilising the barrier and resulting in (7) cytokine/chemokine release and the production of microparticles. This pro-inflammatory state of the endothelial cells, coupled with increased permeability, triggers an inflammatory cascade that disrupts the neurovascular unit by loosening tight junctions, (8) causing pericyte dysfunction and retraction of astrocyte end feet. This non-functional BBB cannot adequately protect the brain. APC, activated protein C; EPCR, endothelial protein receptor C; ICAM-1, intercellular adhesion molecule 1; iRBC, infected red blood cell; NF-κB, nuclear factor kappa-light-chain-enhancer of activated B cells; PAR1, protease-activated receptor 1; Rho A, ras homolog family protein A; S1P, sphingosine-1-phosphate; ZO-1, zonula occludens protein 1.

The human vasculature permeates the entire body, allowing for the rapid transport of oxygenated blood and removal of carbon dioxide. The vascular architecture varies in size and complexity, ranging from large single arteries to the microscopic, highly branched capillary beds (Tortora, and Derrickson, 2016). *P. falciparum*-iRBCs preferentially sequester on the endothelia of the smallest vessels, i.e. capillaries and post-capillary venules, leading to localised occlusions and inflammation (Aikawa, 1988; Grau et al., 2003; Silamut et al., 1999; Taylor et al., 2004). The endothelia can be described as discontinuous, fenestrated or continuous (Fig. 1A). Organs such as the liver, spleen and bone marrow have discontinuous endothelia, in which the endothelial cells have large gaps of approximately 50-180 nm that allow for passage of macromolecules and even red blood cells (RBCs) (Barnhart and Lusher, 1979; Michel, 1996; Snoeys et al., 2007; Stan, 2013; Wisse et al., 2008; Yazdani et al., 2019). The fenestrated capillary beds of organs, such as the kidney, those surrounding endocrine glands and those of intestinal villi and the pancreas contain pores that are approximately 60-80 nm in diameter, which increase the rate of exchange between the intravascular and extravascular compartments. These pores allow for easier transport of water, proteins and larger molecules such as hormones (Jourde-Chiche et al., 2019; Satchell and Braet, 2009). Unlike the capillaries that form the BBB, the brain choroid plexus capillaries are also fenestrated, allowing for the secretion of cerebrospinal fluid (Solár et al., 2020). A continuous endothelium is a key feature of the BBB, in which endothelial cells form tight junctions and are supported by pericytes and astrocytes to precisely regulate the passage of molecules between the bloodstream and the underlying parenchyma (Abbott et al., 2006; Armulik et al., 2010). In contrast to fenestrated and discontinuous endothelia, continuous endothelia lack pores or gaps and exert tighter control over the transport of molecules and cells (Wolburg and Lippoldt, 2002). The absence of pores means that transport is largely transcellular (via transcytosis), although immune cells can still migrate through cellular barriers (Begley and Brightman, 2003; Schinkel, 1999).

## The BBB

The endothelial cells of the cerebral microvasculature lack pores and are capable of forming tight junctions via proteins such as claudin-5 (CLDN5), ZO-1 and occludin (Wolburg and Lippoldt, 2002) (Fig. 1A). Owing to the high metabolic rate of the human brain (Kuzawa et al., 2014), the transport of glucose and other metabolites is essential and can either be passive through facilitative diffusion via glucose transporters (GLUTs), or active through Na^+^/glucose cotransporters, such as SGLTs (reviewed in Joost and Thorens, 2001; Wright et al., 2011). Other mechanisms, such as clathrin-dependent or -independent transcytosis, are employed to transport lipids, other small molecules and nutrients (Pifferi et al., 2021), whereas regulating the transport of potentially harmful substances in and out of the brain (efflux) is mediated by molecules such as P-glycoprotein (Schinkel, 1999). The endothelial cells of the human BBB are supported by pericytes and astrocytes. Pericytes contribute to barrier function by secreting products that assist the distribution of tight junctions, polarisation of astrocytes and clearance of toxins (Armulik et al., 2010; Ma et al., 2018). Astrocytes are amongst the most abundant cell type in the brain and express receptors such as GLUT-1 (SLC2A1), P-glycoprotein, aquaporin 4 (AQP4) and K^+^ channels (Fig. 1B) (Satoh et al., 2007; Seifert et al., 2018; Tower and Young, 1973). These three cell types work together to restrict access to the brain and to enhance the removal of substances harmful to the brain. Pericytes and astrocytes also contribute to endothelial function, support the transport of molecules across the BBB and help maintain brain homeostasis (reviewed in Segarra et al., 2021).

## Investigating the BBB in health and disease

**Fig. 2.**
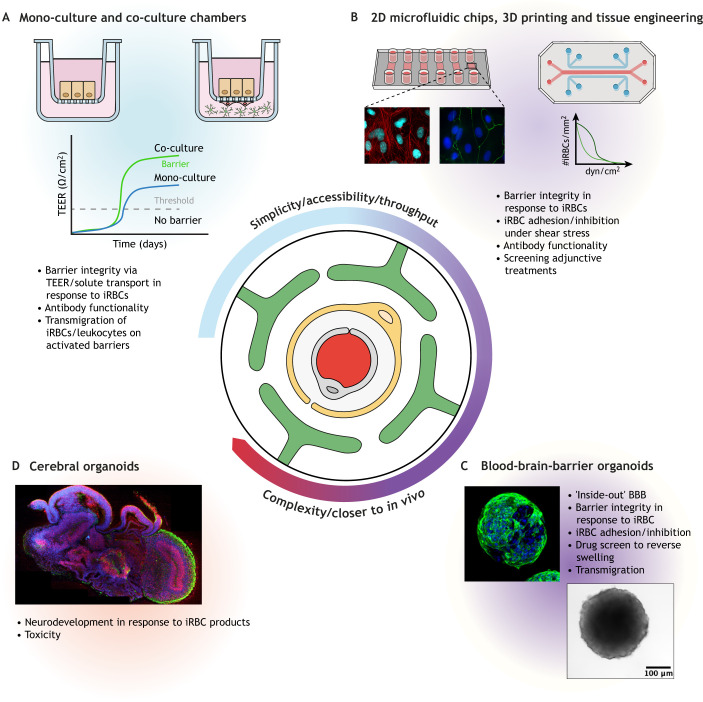
**Summary of *in vitro* models of the brain and BBB.** This diagram summarises the increasing complexity of the models and indicates the possible experimental approaches to interrogate the mechanisms via which *P. falciparum*-infected red blood cells disrupt the BBB and to identify and test potential adjuvant therapeutics. (A) Boyden chambers with mono-/co-culture systems allow for the measurement of barrier integrity in response to iRBC exposure. By activating the endothelial layer, transmigration of iRBCs/leukocytes is measurable. (B) The vasculature is under constant fluid flow and this can be mimicked using 2D or 3D microfluidic chips. Pumps generate shear stress recapitulating that found within the microvasculature. Adhesion under flow conditions can be quantified, as can the effect of antibodies against the parasite-derived *Pf*EMP1. To assess barrier permeability, TEER and solute transport using fluorescent tracers can also be measured. Alterations of endothelial proteins such as actin (red) or the tight-junction protein ZO-1 (green) can be visualised via immunofluorescence. (C) The complexity of the BBB model can be increased by generating BBB organoids. These consist of astrocytes, pericytes and an outer layer of endothelial cells. This allows for the generation of a functional BBB and adhesion of iRBCs. Owing to the induction of swelling by specific iRBCs, which are capable of entering and migrating into the organoids, they also serve as a model for the investigation of neuroprotective agents. (D) The most complex model is the cerebral organoid. Although lacking endothelial cells/vascularisation, cerebral organoids offer a unique opportunity to investigate neurodevelopment in response to parasite exposure and the toxicity of parasite-derived products. BBB organoid and primary human brain endothelial monolayer images courtesy of Y.A., University of Copenhagen. Cerebral organoid image ©IMBA reused with permission (Lancaster et al., 2013). This image is not published under the terms of the CC-BY license of this article. For permission to reuse, please see Lancaster et al. (2013). BBB, blood–brain barrier; iRBC, infected red blood cell; TEER, transepithelial electrical resistance; ZO-1, zonula occludens protein 1.

A healthy, intact BBB not only limits the movement of solutes from the blood stream to the underlying parenchyma, but it also serves to protect the brain from toxins and pathogens. This highly selective barrier therefore poses a challenge when treating neurodegenerative diseases (e.g. Alzheimer's disease), brain cancers (glioblastoma multiforme) or brain-resident infections such as cerebral malaria or Lyme neuroborreliosis. In cerebral malaria, BBB dysfunction and inflammation can be triggered by sequestration of iRBCs or as a response to parasite-derived products such as histones or free haemazoin (Brown et al., 1999; Gillrie et al., 2012; Prato et al., 2011). As the barrier degrades, the underlying pericytes and astrocytes become activated, further destabilising the barrier (Fig. 1B) (Conroy et al., 2010; Deininger et al., 2000; Rustenhoven et al., 2016; Yeo et al., 2008). Whether direct contact via *Pf*EMP1 alone drives the pathology or it works in combination with the action of secretory factors or parasite by-products remains to be determined. Therefore, a model to investigate the BBB *in vitro* is crucial for furthering our mechanistic understanding and for the identification of chemotherapeutics. A number of cell-derived *in vitro* models are available, ranging from simple cellular monolayers to highly complex cerebral organoids (Fig. 2, Table 1). Here, we discuss their benefits and limitations, particularly in the context of cerebral malaria research.

**Table 1.**
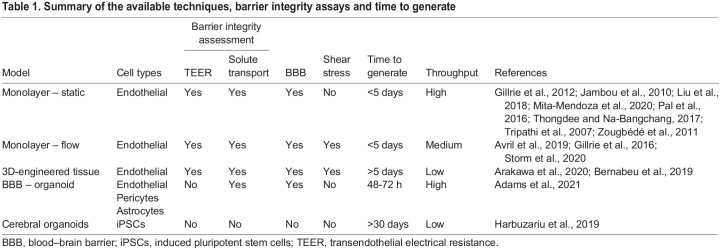
Summary of the available techniques, barrier integrity assays and time to generate

## Cell lines for BBB studies

To correctly model the BBB *in vitro*, researchers must use the appropriate cell lines. A number of primary and immortalised endothelial and astrocyte cell lines are commercially available for such studies, and many have been well characterised, for example, the immortalised human cerebral microvascular endothelial cell lines hCMEC/D3 or HBEC-5i (Dorovini-Zis et al., 1991; Helms et al., 2016; Weksler et al., 2005). The hCMEC/D3 cell line is from a single donor and is used by many laboratories to investigate the BBB in response to neurodegenerative disease and diseases caused by infectious pathogens, such as cerebral malaria (Adams et al., 2021; Adiutori et al., 2021; Jambou et al., 2010; Ma et al., 2018; Okura et al., 2014; Sabiiti and May, 2012; Santiago-Tirado et al., 2019; Vu et al., 2009). The HBEC-5i cell line has also been used for *P. falciparum* research (Adams et al., 2014; Claessens et al., 2012; Wassmer et al., 2005, 2006); however, this line is derived from multiple donors and has an activated profile, which is characterised by a proinflammatory and procoagulant state when the cells are in a resting phase, and which results in higher levels of ICAM-1 and reduced EPCR expression (Dorovini-Zis et al., 1991; Puech et al., 2018; Weksler et al., 2005). Additional immortalised cell lines are available, although these are derived from malignancies, e.g. the astrocyte cell line M059K, which is derived from malignant glioblastoma and might have altered receptor expression profiles compared to normal healthy cells (Liu et al., 2011). Primary cerebral endothelial cells, pericytes and astrocytes are commercially available; however, these may prove to be costly and their batch-to-batch variation may cause issues with reproducibility. One approach to circumvent the issues with commercial primary cells is the use of induced pluripotent stem cells (iPSCs) (reviewed in Workman and Svendsen, 2020). Similar to embryonic stem cells, iPSCs can be reprogrammed to differentiate into any cell type in the human body, allowing for the generation of appropriate cell types when needed (D'Aiuto et al., 2014; Takahashi et al., 2007). As iPSCs are generated from adult cells, they are more readily available, and their differentiation into neuronal cells *in vitro* avoids the use of highly invasive procedures to harvest these cells. A number of laboratories are using iPSCs to generate their models of the BBB, ranging from monolayers to complex cerebral organoids (Camp et al., 2015; Harbuzariu et al., 2019; Kim et al., 2019; Lancaster et al., 2013; Martins Gomes et al., 2019); however, only one group to date has published a study using iPSCs specifically for malaria research (Harbuzariu et al., 2019).

## Monolayers

The simplest model of the BBB is the cellular monolayer method that uses Boyden chambers or culture inserts (Fig. 2A). This model allows for the assessment of barrier integrity and function. Different cell types, from cerebral vasculature (e.g. hCMEC/D3), primary human brain microvascular endothelial cells (HBMECs) or the murine bEnd.3, are grown *in vitro* as simple monolayers on porous cell culture inserts (Borges et al., 1994; Hartz et al., 2010; Weksler et al., 2005; Yang et al., 2017). A voltohmmeter allows for the measurement of the trans-endothelial electrical resistance (TEER) in this system, whereby a high TEER (Ω.cm^2^) correlates with increased barrier integrity, which is the key BBB feature that can be tracked in this model. Researchers can test how different interventions and factors affect barrier integrity, as the TEER decreases in response to barrier disruption. The addition of compounds such as vascular endothelial growth factor (VEGF) acts as a positive control for barrier disruption (Argaw et al., 2009; Proescholdt et al., 1999; Weis and Cheresh, 2005). This method has been used to investigate the invasive and barrier-disrupting properties of bacterial and viral pathogens, such as *Streptococcus*, *Mycobacterium tuberculosis* and, more recently, severe acute respiratory syndrome coronavirus 2 (SARS-CoV-2) (Jain et al., 2006; Kim et al., 2017; Zhang et al., 2021). In malaria research, this method has been used to investigate how parasite-derived products or iRBC binding affect barrier integrity. This was done by co-incubating endothelial cells in the upper chamber with *P. falciparum* isolates or recombinant proteins (Avril et al., 2019; Gillrie et al., 2012; Jambou et al., 2010; Mita-Mendoza et al., 2020; Moxon et al., 2020; Oggungwan et al., 2018; Thongdee and Na-Bangchang, 2017; Tripathi et al., 2007). Kinetic studies that follow the disruption and repair of barrier integrity over time in response to *P. falciparum* exposure (Avril et al., 2019; Gillrie et al., 2016) or those that measure the efficacy of compounds designed to rescue barrier integrity (Liu et al., 2018; Storm et al., 2020) can also be investigated using the Boyden chamber method. Alternatively, barrier integrity can be investigated using specialised slides printed with electrodes (Avril et al., 2019; Gillrie et al., 2016). Monolayers are the least complex, most readily available methods for BBB investigations, but lack the vital components of the *in vivo* barrier, namely, astrocytes and pericytes. This limits the usefulness of the monolayer model for the investigation of iRBCs or iRBC-derived products to endothelial cells only.

## Co-cultures including pericytes and/or astrocytes

*In vivo*, pericytes and astrocytes support the endothelial cells of the BBB by contributing to the formation of tight junctions and enhancing barrier integrity (Armulik et al., 2010; Daneman et al., 2010; Michinaga and Koyama, 2019; Ronaldson and Davis, 2012). This is particularly important, as many cultured human brain microvascular cell lines do not generate high TEER levels (reviewed in Helms et al., 2016). The addition of pericytes and astrocytes can achieve a significant increase in TEER levels (Cho et al., 2017; Hatherell et al., 2011; Urich et al., 2012).

The combination of endothelial cells, pericytes and/or astrocytes into a co-culture system allows for not only the investigation of cellular responses, but also the determination of the efficacy of compounds designed to protect or rescue barrier function (Liu et al., 2018; Storm et al., 2020).

## BBB dysfunction – tight junctions or aberrant transport?

The BBB can be disrupted in two ways, either by loss of tight junction integrity leading to paracellular leakage or by aberrant transport of molecules across the barrier (reviewed in Salimi and Klein, 2019). How a pathogen disrupts the BBB can influence how we measure barrier integrity and interpret the results. The exact mechanism of how iRBCs induce barrier and endothelial dysfunction in cerebral malaria is poorly understood. Early work using monolayers demonstrated barrier dysfunction by reduced TEER and induction of NF-κB, indicating a proinflammatory response (Tripathi et al., 2007, 2009). Other studies have also identified interactions with thrombin and increased fibrin deposition, which may contribute to endothelial dysfunction (Gillrie et al., 2016; Moxon et al., 2013). Assessing tight junctions using TEER is a routine method for assessing barrier integrity; however, resistance levels measured from endothelial cells are generally lower than those from epithelial cells, and experimental conditions such as cell type, insert type, medium viscosity, temperature and method to measure impedance all contribute to the results (Srinivasan et al., 2015). Additionally, not all disruption of the BBB is driven by damage to tight junctions or loss of tight-junction-protein expression as such, and there has been some debate about the validity of TEER and how it can be directly translated into tight-junction and barrier integrity. Indeed, some researchers suggest that use of fluorescently labelled tracers, for example FITC-conjugated dextran sulphates in the 4-150 kDa size range, can better interrogate the permeability of the barrier. Not only does quantification of these tracers from the basal compartment of the culture chamber allow researchers to measure paracellular disruption, microscopic or flow cytometric analysis would allow for quantification of cellular uptake (Chanthick et al., 2016; Helms et al., 2016). New research has recently highlighted the role aberrant transport may play in cerebral malaria. Jin and colleagues reported increased albumin uptake in addition to reversible oedema in the murine model (Jin et al., 2022). This oedema is comparable to that seen in magnetic resonance imaging (MRI) scans of cerebral malaria patients, and the authors indeed report similarities in the human subjects. These new exciting data underpin the need for more studies into the BBB during cerebral malaria and suggest that loss of tight junctions may not be the only driver of dysfunction.

## BBB under flow conditions

The circulatory system is under constant fluid flow, generating shear stress that assists in the polarisation of cells, receptor expression and mechanotransduction (Butler, 2016; Wojciak-Stothard and Ridley, 2003). The shear stress mediated by blood flow assists in the upregulation of multiple endothelial cell receptors used by the malaria parasite for adhesion (Chiu and Chien, 2011; Jun et al., 2009; Nagel et al., 1994). Furthermore, receptors involved in inflammatory responses, such as integrins, become activated under shear stress compared to static conditions (Chen et al., 1999; Shyy and Chien, 2002).

Modelling the BBB under fluid flow more closely mimics the conditions *in vivo*. Advances in microfluidics have led to the ‘organ-on-a-chip’ models for specific organs like the brain (Fig. 2B). By fabricating channels and seeding commercially sourced or iPSC-derived endothelial cells, pericytes and astrocytes into the channels of the chip, a barrier can be formed that mimics the morphology of the microvasculature. Fluid then flows along the channels of the chip, mimicking blood flow. Micropatterning allows for the manufacture of simple linear shapes (Brown et al., 2019; Deosarkar et al., 2015; Tang et al., 2018) or more complex grids (Arakawa et al., 2020; Bernabeu et al., 2019), and seeding on these scaffolds even changes the cellular architecture to more closely mimic that of capillary beds *in vivo* (Oh et al., 2017).

Bio-engineered vessels have primarily been utilised for testing cancer and neurodegenerative disease treatments, but have recently supported studies into the adhesion of *P. falciparum*-iRBCs (Arakawa et al., 2020; Bernabeu et al., 2019; El-Ali et al., 2006; Esch et al., 2015).

A simpler approach to studying the effects of fluid flow on the BBB is the use of commercially available microslides that recapitulate a defined set of shear stresses. Although the bio-engineered vessels allow for more creativity with regards to complex geometry (grid shape or bifurcated channels), the commercial solution using single linear channels in parallel to mimic blood vessels allows for greater reproducibility and does not require specialised equipment and/or reagents needed to manufacture them in-house. By seeding microvascular endothelial cells onto the slides or chips, it is possible to mimic blood flow and pass iRBCs over resting or activated endothelial cells to measure the level and efficiency of iRBC sequestration (Cooke and Nash, 1995; Cooke et al., 1994). It is also possible to purchase more complex geometries mimicking *in vivo* vascular beds, although these chips have not yet been used for malaria research (Khor et al., 2018; Pradhan et al., 2018; Smith et al., 2014). The fact that microslide experiments only require small volumes means that experiments using precious serum samples from patients are possible. This allows for investigations into the adhesive capabilities of iRBCs, localisation of surface receptors via immunofluorescence, antibody specificities and adjunctive treatments (Avril et al., 2019; Dormeyer et al., 2006; Gillrie et al., 2016; Saiwaew et al., 2017). For example, researchers can address whether and how a specific antibody is capable of inhibiting or reversing adhesion (Lennartz et al., 2015; Mustaffa et al., 2017). The introduction of fluid flow takes the chip assay a step closer to *in vivo* conditions. However, this model, like the previously discussed monolayer one, lacks the cellular complexity found within the human body. Both pericytes and astrocytes contribute to the strength of the BBB, and the expression level of the receptors to which iRBCs can adhere may be upregulated or downregulated in the multi-cell *in vivo* barrier compared to their levels in the *in vitro* monolayer (Urich et al., 2013). This may over-report the level of binding whilst under-reporting the effect on tight junctions. There are commercial solutions to this, in which the component cells can be added to distinct channels with a porous membrane separating the astrocytes and pericytes from the endothelial chamber, which is exposed to shear stress (Brown et al., 2019); however, they have not yet been utilised for malaria research.

## Organoid models

*In vitro* models to study the human brain and BBB have undergone a seismic shift in the past decade since the development of advanced cerebral organoid cultures (Fig. 2C,D). These offer a reproducible means to investigate neurodevelopment *in vitro* (Lancaster et al., 2013). The organoids self-assemble and are capable of mimicking the three-dimensional (3D) structure and complexity of the *in vivo* tissue (Lancaster and Knoblich, 2014; Lancaster et al., 2013). Using human iPSCs, cellular aggregates are directed towards differentiation, and cellular heterogeneity is achieved through various conditioned media, resulting in cerebral organoids that recapitulate the foetal developing brain (Camp et al., 2015). Although these organoids are highly complex and include multiple cell types, there are limited reports of vascularised cerebral organoids, with some being human-murine hybrids or containing endothelial cells derived from iPSCs, which may be misidentified (Ahn et al., 2021; Cakir et al., 2019; Lu et al., 2021; Mansour et al., 2018; Shi et al., 2020). Vascularisation improves the long-term growth of organoids and limits necrosis (Mansour et al., 2018); however, such experiments are challenging, not easily transferable and not feasible for most laboratories. Although vascularised cerebral organoids would be an advantageous model system for studying cerebral malaria, these are not yet feasible.

A recent publication from the Lancaster laboratory reports the successful generation of choroid plexus organoids. These organoids mimic the highly selective blood–cerebrospinal fluid barrier (Pellegrini et al., 2020a) and were also found to successfully secrete cerebrospinal fluid. A functional model of the choroid plexus is of particular interest for the investigation of pathogens such as human-infective trypanosomes, *Haemophilus influenzae* or *Neisseria meningitidis*, which interact with or gain entry into the brain via the choroid plexus (Herold et al., 2021; Mogk et al., 2014; Wegele et al., 2020). Although the choroid plexus is typically not a site of sequestration by malaria parasites, recent work by Barrera and colleagues reported the accumulation of CD8^+^ T cells within the choroid plexus in fatal cases of cerebral malaria amongst Malawian children (Barrera et al., 2019). The choroid plexus organoid thus presents a unique opportunity to investigate the role of CD8^+^ T cells in a human-derived model and further demonstrates the immense potential organoid technologies have for cerebral malaria research.

A simpler type of BBB organoids has been successfully developed (Urich et al., 2013). These organoids are 3D aggregates of human primary brain endothelial cells, primary pericytes and primary astrocytes, and therefore recapitulate the cellular composition of the BBB. However, unlike the *in vivo* BBB, the endothelial cells are present on the surface of the aggregate. Owing to the presence and accessibility of endothelial cells, these organoids support the adhesion of iRBCs, possess functional tight junctions, and have proven useful for drug discovery and identification of BBB-permeant drugs and therapeutics (Adams et al., 2021; Cho et al., 2017; Kurbegovic et al., 2021). BBB organoids range in complexity from the original ones containing three cell types – astrocytes, pericytes and endothelial cells (Urich et al., 2013) – to more recent iterations composed of six or more cell types – primary astrocytes, pericytes and endothelial cells, plus iPSC-derived microglia, oligodendrocytes and cortical neuronal cells (Sokolova et al., 2020; Urich et al., 2013). Unlike cerebral organoids, these BBB organoids self-assemble within 48 h and can be generated in 96-well plates, making them compatible with high-content-screening systems. A potential drawback of this model is the lack of fluid flow; however, this can be addressed by using multi-well chips to house the organoids and allow for fluid flow that mimics the shear stress found in the microvasculature (Lim and Park, 2018). Alternatively, a number of newly developed commercial solutions are available. For example, MIMETAS offers modified 96-well plates adapted to hold media reservoirs (OrganoPlate^®^ Graft). Organoids can be grown in these plates, and shear stress is generated by the flow of media from one reservoir to another. Another commercial solution is the use of ibidi™ µ-pattern slides on which organoids can be grown in well-defined patterns via tethering to RGD-peptide domains printed onto the microslide. Cells only grow at the tether sites and a microfluidic pump generates shear stress. These µ-pattern slides, which have been commercially available from 2021, have been shown by the manufacturer to support single-cell-type cancer spheroid growth, not multiple-cell-type-derived organoids. A 3D-perfusion solution is offered by companies such as ibidi™ and QuasiVivo, which uses chips or chambers that allow for shear stress to be generated by a microfluidic pump (Azimi et al., 2020). However, none of these solutions have been reported in the literature for use with cerebral malaria research, or indeed any infectious disease. There is also the added cost of using a proprietary system, such as the specialised 96-well plates, that would need to be considered when choosing a model for BBB research.

## Organoids and cerebral malaria

Cerebral organoids have been embraced very quickly by researchers investigating the effects of pathogens such as the Zika virus (Qian et al., 2016), the Japanese encephalitis virus (Zhang et al., 2018) and *Toxoplasma gondii* (Seo et al., 2020). Impressively, despite the complexity of producing organoids, a large number of peer-reviewed articles on SARS-CoV-2 infection of cerebral organoids were published within the first 24 months of the identification of the virus in November 2019 (a selection can be found here: McMahon et al., 2021; Pellegrini et al., 2020b; Ramani et al., 2021). In contrast, only two peer-reviewed articles report the use of cerebral or BBB organoids for malaria research (Adams et al., 2021; Harbuzariu et al., 2019). Harbuzariu et al. (2019) exposed 20- to 40-day-old cerebral organoids to free haem for 8 h and reported alterations in cellular proliferation and apoptosis. However, these organoids lacked endothelialisation, and the absence of a functional BBB restricted the ability of the organoids to not only inhibit the passage of toxic molecules, but also actively remove toxins (efflux) from the brain. Therefore, the negative effects reported may be exacerbated by the lack of a functional BBB. The studies using this model do, however, show that the negative effects reported can be attenuated by the addition of neuregulin-1 (NRG1) and this is similar to murine studies (Liu et al., 2018; Solomon et al., 2014). To our knowledge, our own group's recent study of iRBCs in a BBB-organoid model is the only paper published to date that used a functional BBB comprising all three cell types (Adams et al., 2021). In this study, we exposed BBB organoids comprising endothelial cells, pericytes and astrocytes to multiple distinct *P. falciparum*-iRBCs and showed that they not only support the adhesion of iRBCs, but also elicit differential responses depending on the parasite isolate to which they were exposed. Parasite isolates associated with cerebral malaria were shown to selectively disrupt the BBB and induce significant swelling of the exposed organoids, whereas exposing the organoids to iRBCs infected with non-cerebral malaria isolates or to non-infected RBCs did not (Adams et al., 2021). The identification of differential disruption of the BBB underlines the need for future mechanistic studies that would eventually identify new adjunctive treatments to reduce or abrogate the effects of cerebral malaria.

Unlike the monolayer models that routinely use TEER to measure barrier integrity in real-time, organoids have routinely been assessed for their barrier function using fluorescently conjugated molecules (Adams et al., 2021; Cho et al., 2017; Kumarasamy and Sosnik, 2021; Urich et al., 2013). The current literature reports a single barrier timepoint, but kinetic studies are possible with access to automated microscopes to track influx of dyes over time, such as in the protocols currently used for tumour spheroid analysis (Kessel et al., 2017; Monjaret et al., 2016). Real-time TEER measurements are possible using micro-electrode probes (Cakir et al., 2019), although this is more technically challenging than the methods used for traditional TEER measurements or the use of fluorescence conjugates, and has yet to be employed in malaria research.

## Conclusions and future directions

The malaria parasite is capable of selectively disrupting the BBB and contributing to brain swelling, which is frequently fatal (Seydel et al., 2015). Amongst those surviving this serious complication, approximately 25% of cerebral malaria survivors are left with long-term neurological sequalae (Idro et al., 2016; John et al., 2008). The essential role of the BBB in cerebral malaria is well documented, but the underlying mechanisms are still incompletely understood. Numerous tools are at the disposal of malaria researchers to study the impact of iRBCs on the BBB. The choice of model will depend on the level of access researchers have to the cells and the equipment needed. Static monolayer models are arguably the most accessible, although organ-on-a-chip and organoid techniques are quickly evolving and becoming more available (Fig. 2). The recent advances in cerebral and BBB organoid cultures mean that we now have the *in vitro* models necessary to investigate what happens not only at but also beyond the BBB. The current cerebral organoid models, which closely resemble the developing brain, could prove very useful for studies into the effect of placental malaria on the developing human brain. In the near future, additional cell types could be added to the BBB organoid model to render it immunocompetent and to drive studies into the immune responses to malaria at the BBB and beyond. By further improving our understanding of the interactions between iRBCs and the BBB, we should be able to develop adjunctive treatments to abrogate the effects of adhesion and develop anti-disease vaccines to reduce the impact of cerebral malaria and improve the outcomes for survivors.
